# Time-varying exposure to food retailers and cardiovascular disease hospitalization and mortality in the netherlands: a nationwide prospective cohort study

**DOI:** 10.1186/s12916-024-03648-w

**Published:** 2024-10-08

**Authors:** Maria Gabriela M. Pinho, Yvonne Koop, Joreintje D. Mackenbach, Jeroen Lakerveld, Mariana Simões, Roel Vermeulen, Alfred J. Wagtendonk, Ilonca Vaartjes, Joline W. J. Beulens

**Affiliations:** 1 Copernicus Institute, Utrecht University, Utrecht, The Netherlands; 2 Amsterdam UMC, Location Vrije Universiteit, Amsterdam, The Netherlands; 3 Julius Center for Health Sciences and Primary Care, University Medical Center Utrecht, Utrecht, The Netherlands; 4 Amsterdam Public Health Research Institute, Amsterdam, The Netherlands; 5 Upstream Team, www.upstreamteam.nl, Amsterdam, The Netherlands; 6 IRAS, Utrecht University, Utrecht, The Netherlands; 7 Amsterdam Cardiovascular Sciences Research Institute, Amsterdam, The Netherlands; 8 Dutch Heart Foundation, The Hague, The Netherlands

**Keywords:** Cardiometabolic health, Exposome, Food retail environment, Longitudinal study, Prevention

## Abstract

**Background:**

Very few studies to date investigated the prospective association of changes in exposure to the food environment with cardiovascular disease (CVD) risk. We aim to explore if time-varying exposure to the food environment was associated with hospitalization and mortality due to total and specific types of CVD in The Netherlands.

**Methods:**

In this prospective cohort study, 4,641,435 Dutch adults aged 35 + years who did not change residence in 2002–2018 were identified through registry data. Exposure to the food environment was defined as time-varying Food Environment Healthiness Index (FEHI) scores (range: − 5 to 5) and time-varying kernel density of specific food retailers (e.g., fast food outlets, supermarkets) around the home location between 2004 and 2018. The main outcome measures were hospitalization and mortality due to overall CVD, stroke, HF, and CHD occurring between 2004 and 2020, based on hospital and death registries.

**Results:**

In Cox regression models, each unit increase in the FEHI was associated with a lower hospitalization and mortality of CVD (hospitalization hazard ratio (HR_h_) = 0.90 (0.89 to 0.91), mortality hazard ratio (HR_m_) = 0.85 (0.82 to 0.89)), CHD (HR_h_ = 0.88 (0.85 to 0.91), HR_m_ = 0.80 (0.75 to 0.86)), stroke (HR_h_ = 0.89 (0.84 to 0.93)), HR_m_ = 0.89 (0.82 to 0.98)), and HF (HR_h_ = 0.90 (0.84–0.96), HR_m_ = 0.84 (0.76 to 0.92)). Increased density of local food shops, fast food outlets, supermarkets, and convenience stores and decreased density of food delivery outlets and restaurants were associated with a higher risk of CVD, CHD, stroke, and HF hospitalization and mortality.

**Conclusions:**

In this observational longitudinal study, changes in exposure to a healthier food environment over 14 years were associated with a risk reduction in CVD hospitalization and mortality, in particular in urbanized areas and for younger adults and those with higher incomes.

**Supplementary Information:**

The online version contains supplementary material available at 10.1186/s12916-024-03648-w.

## Background

Cardiovascular diseases (CVD) are the leading cause of death worldwide, with more than 17 million deaths in 2017 [1]. Unhealthy dietary behaviors contribute to approximately 55% of CVD deaths [2]. Dietary interventions can effectively reduce CVD risk at the individual level [3], but implementation on a large scale is challenging. Moreover, the sustainability of dietary interventions is limited in the long run [3]. Theory on the ‘nutrition transition’ strongly indicates that the changing food environment—defined as the geographical context where people make food choices—contributed to the shift of population dietary patterns from plant-based diets with unprocessed foods towards current Western diets characterized by high levels of energy-dense, high-sugar and low-fiber foods [4, 5]. Indeed, food environments have changed rapidly in the last few decades [6–9]. For example, from 2004 to 2018, the food environment in the Netherlands saw an increase in the availability of restaurants, food delivery services, a decrease in the availability of local food shops such as bakeries, and a moderate increase in the availability of fast food outlets [6]. These trends differed by neighborhood urbanization and socioeconomic position, such that residents living in more urbanized and poor neighborhoods were increasingly more exposed to unhealthy food outlets as compared to those living in more rural and affluent neighborhoods [6]. Although these long-term changes in the food environment happened simultaneously with the increase in the prevalence of overweight and obesity [6, 10], the incidence of CVD has declined over the past decades [11].

However, the current evidence for the impact of exposures on dietary behaviors and CVD risk is limited. The association between the food environment, dietary behaviors, and CVD risk is mostly based on studies with limited sample sizes and cross-sectional designs. To date, only seven prospective cohort studies have investigated the association of exposure to the food environment with CVD risk, mainly focused on the availability or density of fast food outlets. Five studies showed a slightly increased risk with higher exposure to fast food outlets [12–16] and two studies showed no association [17, 18]. Most studies, however, measured exposure to the food environment only at baseline, assuming a stable exposure over the 1 to 7 years follow-up. Only Lovasi et al. investigated the time-varying presence of fast food outlets or supermarkets in relation to CVD risk and did not detect an association in a relatively small sample of 2939 participants [19]. Moreover, these studies did not account for the co-location of other food outlets which may confound the observed associations between specific food retailers and health outcomes. Therefore, strong evidence for the association of changes in the food environment in relation to CVD risk assessing overall healthiness and exposure to specific food retailers is currently lacking.

The aim of this study was to explore if the time-varying availability of different types of food retailers around the home from 2004 to 2018 were associated with the hospitalization and mortality of total and specific types of CVD outcomes (heart failure (HF), stroke, or coronary heart disease (CHD)) in The Netherlands. We hypothesized that changes in exposure to a healthier food environment over 14 years would be associated with decreased risk of CVD, stroke, HF, and CHD morbidity and mortality among the Dutch general population. We additionally tested if associations were different according to sex, age categories, household income, and neighborhood urbanization levels.

## Methods

### Study design and data sources

This was a nationwide, longitudinal time-varying cohort study using registry data from Statistics Netherlands (Centraal Bureau voor de Statistiek, CBS) and the Dutch Hospital Data. CBS has multiple national registries which are largely compiled on a statutory basis for all residents in the Netherlands. By integrating several of these databases we built an administrative cohort with information on individual characteristics (such as age, sex, migration background, income, and marital status), residential history, and neighborhood characteristics. The cohort was linked to the mortality registry and the Dutch Hospital Data via a unique person pseudonymized identifier.

### Exclusion criteria and study population

**Fig. 1:**
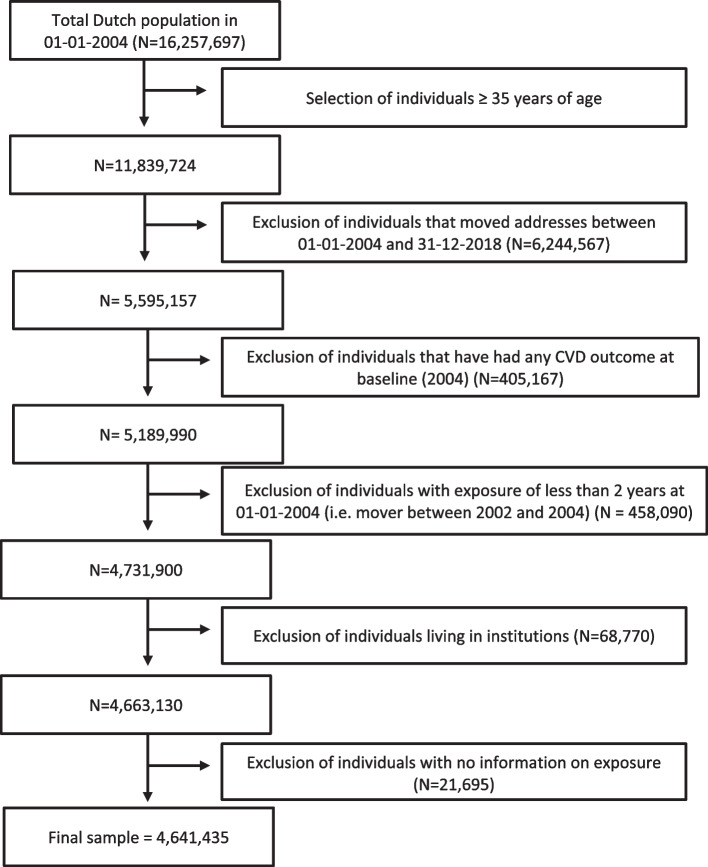
Flow chart showing the cohort selection for analyses by linking time-varying exposure to the food environment to cardiovascular disease hospitalization and mortality in a nationwide study

For this study, we selected all individuals 35 years and older at baseline (01–01-2004), since participants below 35 years of age are assumed to have a very low risk of developing CVD within our study period (*N* = 11,839,724, Fig. 1). To test a potential influence of the residential food environment on CVD outcomes, we assumed that a minimal exposure period of 2 years would be needed at baseline (2004). In addition, this study focuses on inherent changes in the food environment and not changes due to relocation. We therefore excluded individuals who changed addresses between 2002 and 2018 (*N* = 6,244,567). Individuals were also excluded if they were admitted to the hospital for CVD prior to baseline (*N* = 405,167). Individuals were excluded if they were institutionalized (e.g., living in care homes), or if linkage to residential address was not possible as exposure assessment in these situations was not possible or of limited relevance. This resulted in a study population comprising 4,641,435 individuals at baseline.

### Exposure measures

Data on biannual food environment exposures were obtained through the Geoscience and Health Cohort Consortium (GECCO), a nationwide geo data infrastructure [20]. The type and location of food retailers were obtained from a commercial database from Locatus (http://www.locatus/com/en) which has been validated for use in scientific research [21]. Time-varying food environment exposures were assessed through (1) the density of 6 types of food retailers and (2) an overall food environment healthiness index.

First, we used the point location of each food retailer across the Netherlands bi-annually from 2004 to 2018 to calculate the time-varying densities of food delivery outlets, fast food outlets, restaurants, local food shops, supermarkets, and convenience stores. These categories were chosen based on remarkable changes in their availability between 2004 and 2018 [6] (Additional file 1). The kernel density method was used to calculate grid-based kernel density distances to food retailers in a circle with a 1000-m radius using a 25-m grid cell size. Subsequently, these calculated distances were assigned to the corresponding residential location. The 1000-m radii were deemed most appropriate for the Dutch context because it represents a distance that individuals can easily cover by walking or cycling.

Second, we calculated the food environment healthiness index (FEHI). The FEHI integrates the density, distance, and healthiness of food retailers in a given area. The FEHI helps avoid an arbitrary categorization of retail outlets into “healthy” or “unhealthy” and gives a more nuanced indication of the healthiness of the neighborhood food environment based on the overall number of retailers selling food. This index was developed through a Delphi study where 20 Dutch scientists working in the field of nutrition and food environment rated a range of food retailers. Rating was based on the profile of foods that the food retailers offered, that is, the provision of foods that are recommended or discouraged by the Dutch Nutritional Guidelines. Food retailer scores could range from − 5 (less healthy food retailers) to + 5 (healthier food retailers) [22]. In several anonymous rounds, scores and rationales for each outlet were provided, shared among the scientists, and discussed until a consensus was reached. An overview of included food retailers and their respective scores can be found in Additional file 2. The FEHI exposures were created using the kernel density method by applying a spatially weighted averaging method to the scores of each food retailer. We modeled the association of the FEHI with the outcomes per unit increase in the score. Since the FEHI has a total range of 10 points (from − 5 to 5), each unit increase represents a 10% increase. The FEHI index design did not account for the case where no food retailer is available around the home (i.e., there is no healthiness score for having no food retailers near the home). Because of this, it is uncertain if having no food retailers around the home is positive or negative in terms of the healthiness of the food environment. Therefore, if an individual had no food retailers present within 1000 m around the home (< 1%), the median FEHI score value was assigned to them, when calculating the FEHI index. In the case of the density of single food outlets, having no (zero) food retailers present was considered a meaningful value (i.e., one can have zero fast food outlets around their home). Therefore, the exposure variables had no missing values.

### Outcome measures

Hospitalization due to cardiovascular events was retrieved from the *hospital discharge register* managed by Dutch Hospital Data, which contains information on duration and primary and secondary causes of hospital admission, as determined at discharge (classified according to the International Statistical Classification of Diseases and Related Health Problems). The classification of diseases and causes of death before 2013 were converted from ICD version 9 to ICD version 10 (ICD-10). The hospitalization dataset is updated with information received from all hospitals in the Netherlands. Each record contains information of one unique hospital admission, so one person can have multiple records in the hospital admission register. Data on mortality were obtained from the *national cause of death registry*, which contains information on the date and primary cause of death from all deceased persons registered in the Netherlands classified according to ICD-10. The cause of death register receives information on deaths in the Netherlands from the legal reporting system, where a physician issues the cause of death statement and the death certificate.

Individuals were followed from January 1, 2004, until December 31, 2020. An event was defined as hospitalization due to first CVD outcome, or death due to CVD—whichever occurred first. We furthermore evaluated specific CVD events: stroke, HF, and CHD (ICD-10 codes are presented in Additional file 3).

### Covariates

Individual level and neighborhood socio-demographic characteristics were also obtained from CBS using the *population register* dataset (residential address, biological sex, date of birth, migration background, marital status, household composition); the system of social statistical datasets—SSB (household income); and CBS geospatial data (neighborhood urbanization levels). Household income reflects the disposable income of household members standardized by the composition of the household (i.e. number of adults and children—by age) in Euros. Marital status was categorized into divorced/widowed, married/living with a partner, or single. Migration background was defined according to CBS classification which is based on the country of birth of the participants and of their parents (Dutch, Western, non-Western). Western countries of origin are one of the countries in Europe (excluding Türkiye), North America, and Oceania. Non-Western countries of origin are one of the countries in Africa, Latin America, and Asia (including Türkiye). Neighborhood urbanization level is defined by CBS in five categories according to the neighborhood density of addresses per square kilometer: very high urbanization (2500 addresses per km^2^ or more); high urbanization (1500 to 2500 addresses per km^2^); moderate urbanization (1000 to 1500 addresses per km^2^); low urbanization (500 to 1000 addresses per km^2^); no urbanization (less than 500 addresses per km^2^).

### Ethics and privacy

We did not involve patients or the public in the design and conduct of the research.

All data linkages and analyses were performed by researchers with strict authorized access in a secure environment of CBS, and in agreement with the privacy regulations in The Netherlands. To guarantee that outputs could not be traced to individuals, all records and datasets were pseudo-anonymized in the CBS analysis environment. Data linkage from national registries was performed using this pseudonym. Furthermore, all analysis results are evaluated by CBS before publication, to ensure compliance to privacy regulations. In compliance to research regulations concerning the Dutch law on Medical Research in Humans, approval by an ethics committee was not required for the present study.

### Statistical analyses

Descriptive statistics are presented for the study population and for the food environment exposures in 2004 and 2018. All variables are time-varying, except sex and migration background, and therefore, regular proportional hazard assumptions do not apply to them. We tested the proportional hazard assumption for sex and migration and confirmed that the proportional hazard assumption was not violated on these variables, plots are presented in Additional file 4. Thus, we applied extended multivariate time-dependent Cox regression models, in which the exposures and covariates were updated biannually. Follow-up ended at the end of the follow-up period (31–12-2020), at the time of the event, when individuals died from other causes, or when individuals were lost to follow-up, whichever came first. Each CVD outcome was tested in a separate model with each of the exposure measures (i.e., the FEHI index, density of local food shops, fast food outlets, food delivery outlets, restaurants, supermarkets, and mini-supermarkets). Linearity of the associations was tested by assessing a scatterplot, the model residuals’ mean, normality, and homoscedasticity, and evaluating HR deviation using quintiles, and all associations were linear and thus modeled continuously per unit change of the exposure.

Incomplete recording of covariates was assumed to be missing at random and imputed accordingly. Missing data on the household income variable (2.3%) was dealt with using linear interpolation using available income data from other years (2003–2019) for each person with a missing income value, and the mode value was used to impute missing values on the urbanization variables (missing < 1%). Household composition data for 2016 and 2018 was not available because a different type of registration was used by CBS. Therefore, the last observation available (2014) was carried forward to fill in these missing years.

Analyses were a priori adjusted for potential confounding by age, sex, migration background, household composition, income, marital status, and neighborhood urbanization level. Stratified analyses were performed according to sex at baseline, levels of urbanization in 2018; deciles of household income in 2018 (low (< 10th percentile), middle (10–90th percentile) and high (> 90th percentile)) according to CBS categorization) [23]; and age groups (35–49; 50–64; 65 and above at baseline) for the CVD hospitalization outcomes.

To account for the co-location of different types of food retailers we conducted sensitivity analyses. In models where density to a specific food retailer (e.g., fast food restaurants) was the main exposure measure, we additionally adjusted for the kernel density of the five remaining food retailers.

All analyses were conducted in R Statistical Software (version 3.6.2, R Foundation). Results are considered statistically significant if the 95% confidence interval (95% CI) excluded unity. However, our large sample size may detect statistically significant results at very small effect sizes. Therefore, when interpreting our results, we did not rely on statistical significance, but on the clinical or public health relevance of our findings.

## Results

The cohort size was 4,641,435 individuals in 2004 and 4,222,646 in 2018 (Table 1). At baseline mean age of the study population was 54.6 ± 12.6 years, 51.3% were female, 91.2% had no migration background, 74.1% were married, and 62.9% lived in a neighborhood that was not urbanized to moderately urbanized. The median annual disposable household income was €19,790. During 192 months (16 years) of follow-up, 1,473,042 (31.7%) individuals were hospitalized for CVD and 230,191 (5.0%) died due to CVD (Additional file 5). Median time to event (IQR) in months were the following for each outcome: CVD hospitalization 107.8 (52.6, 155.2); CVD mortality 104.1 (49.3, 156.5); CHD hospitalization 92.3 (41.3, 144.1); CHD mortality 87.5 (38.4, 144.8); stroke hospitalization 111.6 (52.3, 157.5); stroke mortality 113.1 (53.3, 160.8); HF hospitalization 91.7 (39.7, 144.9); HF mortality 120.9 (64.3, 165.8). Additional file 6 shows the distribution of the exposure to the food environment in 2004 and 2018. Most remarkable was the reduction on the kernel density of local food shops, which had a median (IQR) of 1.65 (0.44–3.56) in 2004, reducing to 1.08 (0.16–2.56) in 2018. The mean (SD) FEHI was − 0.09 (0.10) in 2004 and − 0.10 (0.11) in 2018. The mean (SD) FEHI was − 0.09 (0.10) in 2004 and − 0.10 (0.11) in 2018. The absolute number of cases per year for each CVD outcome can be found in Additional file 7, indicating that a 10% risk reduction of CVD would result in an estimated absolute reduction of 18,000 cases of CVD hospitalization per year.

**Table 1:** Descriptive characteristics of the cohort in 2004 and 2018

	**01–01-2004**		**01–01-2018**	
	*n*	Mean (sd) or %	*n*	Mean (sd) or %
Age (mean)	4,641,435	54.6 (12.6)	4,222,646	67.0 (11.6)
Sex, female (%)	2,380,101	51.3%	2,191,377	51.9%
Ethnicity				
* Dutch*	4,232,531	91.2%	3,844,191	91.0%
* Western*	200,516	4.3%	178,787	4.2%
* Non-western*	208,388	4.5%	199,668	4.7%
Annual household income (€, median (IQR)		19,790 (15,118–25,999)		29,038 (21,713–37,981)
Marital status				
* Divorced/widowed*	707,460	15.2%	926,595	21.9%
* Married/living with partner*	3,440,257	74.1%	2,882,132	68.3%
* Single*	493,718	10.6%	413,919	9.8%
Neighborhood urbanization levels				
* No urbanization*	1,031,219	22.2%	828,026	19.6%
* Low urbanization*	976,529	21.0%	750,507	17.8%
* Moderate urbanization*	913,321	19.7%	828,168	19.6%
* Strong urbanization*	1,039,058	22.4%	1,068,545	25.3%
* Very strong urbanization*	681,308	14.7%	747,400	17.7%

**Fig. 2:**
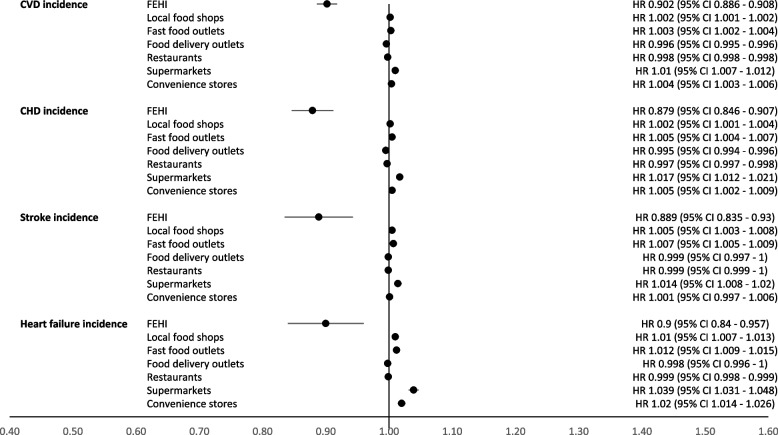
Hazard ratios and confidence for hospitalization due to cardiovascular events in relation to longitudinal exposure to the Food Environment Healthiness Index (FEHI) or exposure to food stores in a 1000-m buffer

**Fig. 3:**
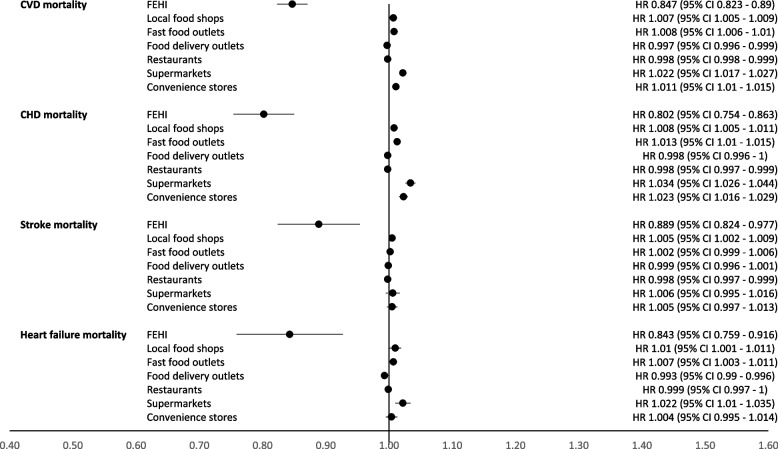
Hazard ratios and confidence for mortality due to cardiovascular events in relation to longitudinal exposure to the Food Environment Healthiness Index (FEHI) or exposure to food stores in a 1000-m buffer

In adjusted Cox regression models, each unit increase in the FEHI index, representing a 10% improvement, was associated with a reduced risk of CVD hospitalization with a hazard ratio (HR_h_) of 0.902 (95% CI: 0.896 to 0.908) (Fig. 2, Additional file 8). Similar associations were observed for hospitalization of CHD (HR_h_ = 0.879 (0.846 to 0.907)); stroke (HR_h_ = 0.889 (0.835 to 0.930)); and HF (HR_h_ = 0.900 (0.840 to 0.957)). Changes in exposure to a healthier food environment, indicated by increases in FEHI, were associated with reduced CVD mortality (HR_m_ = 0.847 (0.823 to 0.890) (Fig. 3, Additional file 9). Again, these associations were similar for CHD mortality (HR_m_ = 0.802 (0.754 to 0.863)), stroke mortality (HR_m_ = 0.889 (0.824 to 0.977)), and HF mortality (HR_m_ = 0.843 (0.759 to 0.916)).

Regarding exposure to changes in the density of individual food retailers, each unit increase in the density of local food shops, fast food outlets, supermarkets, and convenience stores was associated with a higher risk of overall CVD, and specifically CHD, stroke, and HF hospitalization (Figs. 2 and 3). Each unit increase in the density of food delivery outlets and restaurants was associated with a reduced hospitalization for all outcomes. Similar results were observed for CVD, CHD, stroke, and HF mortality (Fig. 3). Additionally, adjusting the models for the broader food environment did not change the results (Additional files 10 and 11).

### Stratified analysis

Associations of food retailer exposures with cardiovascular outcomes were strongest and most consistently associated with outcomes in the highly urbanized areas (Additional file 12). For example, the HR_h_ for CVD hospitalization for FEHI in highly urbanized areas was 0.799 (0.726 to 0.879) and 0.960 (0.940 to 0.980) in non-urban areas. These results were consistent across the different CVD outcomes. However, a higher density of food delivery outlets was associated with lower hospitalization for CVD, CHD, and stroke in high and very high urbanized areas, while it was associated with higher CVD hospitalization in moderate and low-urbanized areas. Higher density of food delivery outlets was associated with lower risk of CVD and CHD mortality in very high urbanized areas only.

Analyses stratified by sex (Additional file 13) suggest that associations were similar for men and women, but analyses stratified by age (Additional file 14) suggest slightly stronger associations for younger (35–64 years) than older individuals (≥ 65 years). The observed CVD risk reduction with increased FEHI was also similar for participants with low and middle incomes, but slightly stronger for high-income neighborhoods (Additional file 15).

## Discussion

Our nationwide study among more than four million Dutch adults showed that a 10% change towards a healthier residential food environment was associated with a 10% to 20% reduced risk of overall CVD, stroke, HF, and CHD hospitalization and mortality. Increasing density of fast food outlets and convenience stores, but also of local food stores and supermarkets, was associated with higher CVD hospitalization and mortality, while higher density of food delivery outlets and restaurants was associated with reduced risks, although effect sizes were small. Associations did not differ by sex, but were somewhat stronger and more consistent in urbanized versus non-urbanized neighborhoods, for younger versus older individuals, and in high versus low- and middle-income individuals.

The results should be viewed in the light of some limitations. CVD hospitalization was based on hospital registrations and mortality based on the primary cause of death, so we may have missed cases. With regard to exposure assessment, we only considered food retailer exposure around the home area while exposure in other relevant areas (e.g., work) would likely have provided a more accurate and comprehensive exposure assessment. Furthermore, our study captures changes in the food environment over 14 years. In this long-term time-period, the incidence of CVD and both individual and environmental risk factors have changed over time, which may distort our findings. However, since we updated exposures and outcomes available in our analyses every 2 years, we do not think this confounded our results to a large extent. Finally, our study likely has remaining uncontrolled confounding, for example, due to lifestyle behaviors such as smoking and physical activity, and also due to psychosocial conditions such as stress and social relations, for which we did not have data. Given this and the observational design of the study, we cannot make strong causal inferences.

This is the first study to show a CVD risk reduction resulting from time-varying increases in the overall healthiness of the residential food environment. Three prospective studies investigated exposure to both healthy and unhealthy food outlets with CVD outcomes of which two observed no associations of both healthy and unhealthy food retailers with CVD outcomes [19, 24] while a third showed that increased exposure to both healthy and unhealthy food stores was associated with an increased risk of cardiometabolic and all-cause mortality [17]. Only the study by Mooney et al. investigated the overall healthiness of the food environment via the proportion of healthy food outlets in a census tract but found no association with the incidence of cardiac arrests [24].

Previous studies found limited evidence for differential associations of the (food) environment with health outcomes across strata of age [18], sex [14, 17, 18], income [18], or urbanization [17]. However, these studies had smaller sample sizes and fixed-time exposures, leading to potentially less power to detect differential associations across strata. Similar findings have been reported for the association of neighborhood drivability and diabetes incidence [25]. Our findings showed that changes in exposure to a healthier food environment were associated with a risk reduction in CVD, with stronger evidence in urbanized areas, among younger adults and those with higher incomes. These differential associations could be explained by the higher range and variety of food outlets present in more urbanized neighborhoods, and the higher ability, habits, and preference to make use of the food environment of younger adults and more wealthy individuals. Indeed, previous research among older adults in the Netherlands found that those with higher income were more likely to consider a healthy and tasteful meal to be important than lower income individuals [26]. The fact that higher income individuals tend to be more health-conscious may partially explain the stronger protective effect of changes to a healthier food environment that were found among this group. Nevertheless, further research is required on how individual’s interact with their food environment.”

The results observed in our and previous longitudinal studies seem to suggest that overall (un)healthiness of the residential food environment is associated with CVD risk, but that associations with specific food retailers are mixed. Indeed, individuals typically use a range of different food retailers [27], and unhealthy foods are purchased from a range of outlets, not just the “classic” unhealthy food outlets such as fast food outlets [28]. This may in part be attributable to changes in the in-store food environment, e.g., in supermarkets, whereby the ratio of healthy and unhealthy options has shifted over time. The slightly increased risk of CVD associated with being exposed to more supermarkets over time may be explained by the overwhelming availability of unhealthy products in Dutch supermarkets (about 80% of the assortment), thereby providing more opportunities for purchasing unhealthy than healthy food [29]. On the other hand, the slightly protective association of restaurants and food delivery services is counterintuitive and should be explored further in future research. It is important to note that the results derived from the FEHI and the individual food retailers cannot be directly compared given the different units of these variables. Nevertheless, it makes sense that the total exposure to all food retailers combined is more strongly related to CVD risk than the exposure to a single food retailer. The directions of the associations with single food retailers are more difficult to explain, although unexpected or null associations have been observed in previous research [30, 31]. In general, previous research indicated that the food environment in the Netherlands is not getting healthier. When looking into the count of individual food retailers, a Dutch study found a 120% increase in the availability of food delivery outlets and a 35% increase in the availability of restaurants. On the other hand, the availability of local food shops decreased by 24% in the same study period [6]. Trends towards a less healthy food environment have also been found in countries such as the USA [8, 32] and the UK [7].

Over a 14-year period, a 10% increase in overall food environment healthiness was associated with a 10 to 20% risk reduction in several CVD outcomes. We consider this risk reduction to be relevant for public health given that this is within the range of relative risks found for other well-known and clinically relevant individual and environmental risk factors. For example, a risk reduction of 31% in the association between a prudent diet and reduced CVD risk [33]; a 8% increased risk of ischemic heart disease at every 10 dB increase in road noise [34]; or a 10% to 15% excess risk of all-cause CVD mortality by long term exposure to air pollution (PM2.5) [35]. In addition to this, as with many environmental exposures, because the reach is very large (i.e., most part of the population is exposed to the food environment), even smaller effect sizes would be relevant at the populational level.

There are some examples of strategies to improve food environment healthiness, such as in the UK and USA where new fast food outlets were banned, with mixed effects on the number of fast food outlets, diet, and obesity [36–38]. Future studies could focus on the combination of policy measures needed to create a sufficient change in the healthiness of food environments that will substantially reduce the burden of CVDs.

## Conclusions

We provide strong evidence that increases in the overall healthiness of the residential food environment over a 14-year period are associated with reduced risk of overall and specific CVD, in particular in urbanized areas and for younger adults and those with higher incomes. Given the high disease burden of CVD and other diet-related chronic diseases, it seems imperative to address the unhealthy food environment as part of a set of population-level interventions that help sustainably diet-related CVDs.

## Supplementary Information

Additional file 1. Description of the characteristics of the food retailers categories used for exposure measures.

Additional file 2. Food outlets description and the scores assigned to them according to the food environment healthiness index (FEHI).

Additional file 3. List of ICD to 9 and ICD to 10 used per outcome.

Additional file 4 Testing the proportional hazard assumption for sex and migration background.

Additional file 5. Cardiovascular disease events at the end of follow to up.

Additional file 6: Distribution of the exposure to the food environment in 2004 and 2018

Additional file 7. Absolute values and percentages of cases per year and total study period (*n*= 4,641,435).

Additional file 8. Hazard Ratios and confidence intervals for Hospitalization for general and specific cardiovascular events in relation to longitudinal exposure to neighborhood food environment or food stores in a 1000 to meter buffer.

Additional file 9. Hazard Ratios and confidence intervals for general and specific cardiovascular mortality in relation to longitudinal exposure to neighborhood food environment or food stores in a 1000 to meter buffer.

Additional file 10. Hazard Ratios and confidence intervals for Hospitalization of general and specific cardiovascular events in relation to longitudinal exposure to neighborhood food environment – models additionally adjusted for the broader food environment.

Additional file 11. Hazard Ratios and confidence intervals for general and specific cardiovascular mortality in relation to longitudinal exposure to neighborhood food environment – models additionally adjusted for the broader food environment.

Additional file 12. Hazard Ratios and confidence intervals for Hospitalization of general and specific cardiovascular events in relation to longitudinal exposure to neighborhood food environment – analyses stratified by neighborhood urbanization.

Additional file 13. Hazard Ratios and confidence intervals for general and specific cardiovascular Hospitalization in relation to longitudinal exposure to neighborhood food environment – analyses stratified by sex.

Additional file 14. Hazard Ratios and confidence intervals for Hospitalization of general and specific cardiovascular events in relation to longitudinal exposure to neighborhood food environment – analyses stratified by age.

Additional file 15. Hazard Ratios and confidence intervals for Hospitalization of general and specific cardiovascular events in relation to longitudinal exposure to neighborhood food environment – analyses stratified by levels of household income.

## Data Availability

Data is not available to unauthorized researchers. All data linkages and analyses were performed by researchers with strict authorized access in a secure environment of CBS. A formal collaboration agreement must be signed between interested parties and CBS in order to access the data.

## Abbreviations

CVD: Cardiovascular diseases
HF: Heart failure
CHD: Coronary heart disease
CBS: “Centraal Bureau voor de Statistiek”—Statistics Netherlands
GECCO: Geoscience and health cohort consortium
FEHI: Food environment healthiness index
ICD: International Classification of Diseases
HR: Hazard ratio
IQR: Interquartile range
CI: Confidence interval
SD: Standard deviation

## Data availability

Data is not available to unauthorised researchers. All data linkages and analyses were performed by researchers with strict authorised access in a secure environment of CBS. A formal collaboration agreement must be signed between interested parties and CBS in order to access the data.
