# Supplementary material for: Time-varying exposure to food retailers and cardiovascular disease hospitalization and mortality in the netherlands: a nationwide prospective cohort study
Source: BMC Med. 2024 Oct 8;22:427. doi: 10.1186/s12916-024-03648-w (PMC11462997; doi:10.1186/s12916-024-03648-w)
Supplement: Supplementary file 1 — Additional file 1. Description of the characteristics of the food retailers categories used for exposure measures. [file 12916_2024_3648_MOESM1_ESM.docx]

**Additional files of ‘Time-varying exposure to food retailers and cardiovascular disease hospitalization and mortality in the Netherlands: A nationwide prospective cohort study**

**Additional file 1**. Description of the characteristics of the food retailers categories used for exposure measures

| **Analytical category** | **Composed of the following food retailers** | **Description of food retailers by main food provision** |
| --- | --- | --- |
| Density of local food shops | Fruit and vegetables store | Fresh produce in general, e.g., potatoes, vegetables and fruit |
|  | Poultry stores | Poultry and other birds |
|  | Butchery | Meat and meat products |
|  | Fish stores | Fish, crustaceans and molluscs |
|  | Bakery | Bread and pastries. Table service is possible, but not be the main store activity |
| Density of fast food outlets | Fast to food outlets | Mostly deep to fried products that are ready for consumption in few minutes after ordering. Usually there is no table service available. |
|  | Grillroom/Shoarma/Kebab shop | Grilled meat meals, shawarma, kebab |
| Density of food delivery outlets | Delivery/take to away | Meals that are not consumed in the store, but are collected or delivered. These retailers can be the same as the restaurant or fast food categories, but also places that only operate delivery. |
| Density of restaurants | Restaurant | Meals a la carte, table service is present |
|  | Restaurant in hotel | Overnight in combination with an la carte restaurant |
|  | Café/restaurant | Provision of both drinks and simple meals |
| Density of supermarket | Supermarket | Wide range of food and non to food products. Store size is at least 150 m² |
| Density of convenience stores | Mini supermarkets | Same as supermarkets but store size is less than 150 m2. This category is composed of stores such as corner shops, night shops, but also smaller versions of chain supermarkets located at strategic places (e.g., Albert Heijn to go). Mini supermarkets selling fresh produce were excluded from this category. |
| Food retailers used to calculate the density to ‘other food retailers’ used as adjustment variables | Nut stores | Nuts |
|  | Turkish/Moroccan supermarkets | Small supermarkets with display of fruit and vegetables at store front |
|  | Ethnic stores | Mainly oriental foods |
|  | Coffee and Tea stores | Coffee and tea |
|  | Bio supermarkets | Biological/organic foods |
|  | Delicacies | Special/luxurious foodstuffs and often also many ready to made products |
|  | Cheese store | Cheese |
|  | Lunchrooms | Lunch meals, unlike restaurants, mainly closed in the evening |
|  | Night shops | Convenience stores with (late) evening and night opening |
|  | Café | Provision of almost exclusively drinks (no meals) |
|  | Pancake restaurant | Restaurant specializing in Dutch pancakes and/or poffertjes |
|  | Ice to cream store | Ice to cream |
|  | Chocolate store | Chocolate |
|  | Pie store | Cakes and pies |
|  | Tobacco store and newsstand | Tobacco, magazines and cadies |
|  | Convenience store at petrol station | Snacks, fast to foods and candies |
|  | Liqueur store | Alcoholic beverages |
|  | Confectionery store | Confectionary and chocolates |
