# Supplementary material for: Time-varying exposure to food retailers and cardiovascular disease hospitalization and mortality in the netherlands: a nationwide prospective cohort study
Source: BMC Med. 2024 Oct 8;22:427. doi: 10.1186/s12916-024-03648-w (PMC11462997; doi:10.1186/s12916-024-03648-w)
Supplement: Supplementary file 2 — Additional file 2. Food outlets description and the scores assigned to them according to the food environment healthiness index (FEHI). [file 12916_2024_3648_MOESM2_ESM.docx]

**Additional files of ‘Time-varying exposure to food retailers and cardiovascular disease hospitalization and mortality in the Netherlands: A nationwide prospective cohort study**

**Additional file 2**. Food outlets description and the scores assigned to them according to the food environment healthiness index (FEHI)

| **Food outlet** | **Description of food outlet by main food provision** | **Scores** |
| --- | --- | --- |
| Fruit and vegetables store | Potatoes, vegetables and fruit | 4.8 |
| Fish stores | Fish, crustaceans and molluscs | 2.8 |
| Nut stores | Nuts | 2.2 |
| Turkish/Moroccan supermarkets | Small supermarkets with display of fruit and vegetables at store front | 2.0 |
| Supermarket | Wide range of food and non to food products. Store size is at least 150 m² | 1.8 |
| Ethnic stores | Mainly oriental foods | 1.5 |
| Coffee and Tea stores | Coffee and tea | 1.3 |
| Bakery | Bread and pastries. Table service is possible, but not be the main store activity | 1.2 |
| Bio supermarkets | Biological/organic foods | 1.0 |
| Poultry stores | Poultry | 0.6 |
| Mini to supermarkets | Same as supermarkets but store size is less than 150 m2 | 0.3 |
| Delicacies | Special/luxurious foodstuffs and often also many ready to made products | 0.2 |
| Butchery | Meat and meat products | -0.4 |
| Restaurant in hotel | Overnight in combination with an a to la to carte restaurant | -0.9 |
| Restaurant | Meals a to la to carte, table service is present | -0.9 |
| Cheese store | Cheese | -1.3 |
| Lunchrooms | Lunch meals, unlike restaurants, mainly closed in the evening | -1.5 |
| Café to restaurant | Provision of both drinks and simple meals | -1.9 |
| Night shops | Convenience stores with (late) evening and night opening | -2.1 |
| Café | Provision of almost exclusively drinks (no meals) | -2.3 |
| Pancake restaurant | Restaurant specializing in Dutch pancakes and/or poffertjes | -3.2 |
| Delivery/take to away | Meals that are not consumed in the store, but are collected or delivered | -3.7 |
| Ice to cream store | Ice to cream | -3.9 |
| Chocolate store | Chocolate | -4.3 |
| Pie store | Cakes and pies | -4.4 |
| Tobacco store and newsstand | Tobacco, magazines and cadies | -4.5 |
| Convenience store at petrol station | Snacks, fast to foods and candies | -4.5 |
| Liqueur store | Alcoholic beverages | -4.6 |
| Confectionery store | Confectionary and chocolates | -4.7 |
| Grillroom/Shoarma/Kebab shop | Grilled meat meals, shawarma, kebab | -4.8 |
| Fast to food outlets | Mostly deep to fried products that are ready for consumption in few minutes after ordering. Usually there is no table service available. | -4.9 |
