# Supplementary material for: Time-varying exposure to food retailers and cardiovascular disease hospitalization and mortality in the netherlands: a nationwide prospective cohort study
Source: BMC Med. 2024 Oct 8;22:427. doi: 10.1186/s12916-024-03648-w (PMC11462997; doi:10.1186/s12916-024-03648-w)
Supplement: Supplementary file 3 — Additional file 3. List of ICD to 9 and ICD to 10 used per outcome. [file 12916_2024_3648_MOESM3_ESM.docx]

**Additional files of ‘Time-varying exposure to food retailers and cardiovascular disease hospitalization and mortality in the Netherlands: A nationwide prospective cohort study**

**Additional file 3**. List of ICD to 9 and ICD to 10 used per outcome

|  | **ICD to 9 codes** | **ICD to 10 codes** |
| --- | --- | --- |
| **Cardiovascular disease** | 391, 392.0, 393 – 434 and 436 - 459 | I00 - I99 |
| **Coronary heart disease** | 410 - 414 | I20 - I25 |
| **Stroke** | 430 – 434 and 436 - 438 | I60 - I69 |
| **Heart failure** | 428 | I50 |
