# Supplementary material for: Time-varying exposure to food retailers and cardiovascular disease hospitalization and mortality in the netherlands: a nationwide prospective cohort study
Source: BMC Med. 2024 Oct 8;22:427. doi: 10.1186/s12916-024-03648-w (PMC11462997; doi:10.1186/s12916-024-03648-w)
Supplement: Supplementary file 5 — Additional file 5. Cardiovascular disease events at the end of follow to up. [file 12916_2024_3648_MOESM5_ESM.docx]

**Additional files of ‘Time-varying exposure to food retailers and cardiovascular disease hospitalization and mortality in the Netherlands: A nationwide prospective cohort study**

**Additional file 5.** Cardiovascular disease events at the end of follow to up

|  | **Hospitalization** | **Mortality** |
| --- | --- | --- |
| Cardiovascular disease (any) | 1 473 042 (31.7%) | 230 191 (5.0%) |
| Coronary heart disease | 322 523 (6.9%) | 65 914 (1.4%) |
| Stroke | 141 379 (3.0%) | 47 545 (1.0%) |
| Heart failure | 72 567 (1.6%) | 34 985 (0.8%) |

Events at end of follow to up 31 to 12 to 2020
