# Supplementary material for: Time-varying exposure to food retailers and cardiovascular disease hospitalization and mortality in the netherlands: a nationwide prospective cohort study
Source: BMC Med. 2024 Oct 8;22:427. doi: 10.1186/s12916-024-03648-w (PMC11462997; doi:10.1186/s12916-024-03648-w)
Supplement: Supplementary file 6 — Additional file 6: Distribution of the exposure to the food environment in 2004 and 2018 [file 12916_2024_3648_MOESM6_ESM.docx]

**Additional files of ‘Time-varying exposure to food retailers and cardiovascular disease hospitalization and mortality in the Netherlands: A nationwide prospective cohort study**

**Additional file 6:** Distribution of the exposure to the food environment in 2004 and 2018

|  |  |  |  | **IQR** | |
| --- | --- | --- | --- | --- | --- |
| **Exposure** | **Mean** | **SD** | **Median** | **Lower quartile** | **Upper quartile** |
| **2004** |  |  |  |  |  |
| FEHI | -0.09 | 0.10 | -0.08 | -0.10 | -0.05 |
| Local food shops | 2.73 | 3.62 | 1.65 | 0.44 | 3.56 |
| Fast food outlets | 1.95 | 3.05 | 1.00 | 0.33 | 2.25 |
| Food delivery outlets | 1.24 | 3.29 | 0.00 | 0.00 | 1.00 |
| Restaurants | 2.77 | 7.99 | 0.88 | 0.14 | 2.24 |
| Supermarkets | 1.01 | 0.89 | 0.84 | 0.32 | 1.49 |
| Convenience stores | 0.38 | 1.15 | 0.00 | 0.00 | 0.15 |
| **2018** |  |  |  |  |  |
| FEHI | -0.10 | 0.11 | -0.08 | -0.12 | -0.06 |
| Local food shops | 1.86 | 2.44 | 1.08 | 0.16 | 2.54 |
| Fast food outlets | 1.78 | 2.76 | 0.93 | 0.27 | 2.09 |
| Food delivery outlets | 2.21 | 5.09 | 0.00 | 0.00 | 2.00 |
| Restaurants | 2.86 | 7.59 | 0.84 | 0.10 | 2.26 |
| Supermarkets | 1.11 | 1.14 | 0.85 | 0.29 | 1.57 |
| Convenience stores | 0.45 | 1.26 | 0.00 | 0.00 | 0.37 |
