# Supplementary material for: Time-varying exposure to food retailers and cardiovascular disease hospitalization and mortality in the netherlands: a nationwide prospective cohort study
Source: BMC Med. 2024 Oct 8;22:427. doi: 10.1186/s12916-024-03648-w (PMC11462997; doi:10.1186/s12916-024-03648-w)
Supplement: Supplementary file 7 — Additional file 7. Absolute values and percentages of cases per year and total study period (n= 4,641,435). [file 12916_2024_3648_MOESM7_ESM.docx]

**Additional files of ‘Time-varying exposure to food retailers and cardiovascular disease hospitalization and mortality in the Netherlands: A nationwide prospective cohort study**

**Additional file 7.** Absolute values and percentages of cases per year and total study period (n= 4,641,435)

|  | **Hospitalization** | | | | **Mortality** | | | |
| --- | --- | --- | --- | --- | --- | --- | --- | --- |
|  | **CVD n(%)** | **CHD n(%)** | **Stroke n(%)** | **Heart Failure n(%)** | **CVD n(%)** | **CHD n(%)** | **Stroke n(%)** | **Heart Failure n(%)** |
| **2004** | 184,258 (4.0) | 49,638 (1.1) | 17,701 (0.4) | 11,624 (0.3) | 29,031 (0.6) | 10,685 (0.2) | 3,063 (0.1) | 5,616 (0.1) |
| **2006** | 153,999 (3.3) | 41,863 (0.9) | 15,058 (0.3) | 9,420 (0.2) | 26,946 (0.6) | 9,216 (0.2) | 3,295 (0.1) | 5,115 (0.1) |
| **2008** | 151,453 (3.3) | 38,841 (0.8) | 13,577 (0.3) | 8,411 (0.2) | 25,188 (0.5) | 8,180 (0.2) | 3,382 (0.1) | 4,799 (0.1) |
| **2010** | 159,786 (3.4) | 36,106 (0.8) | 14,071 (0.3) | 8,083 (0.2) | 24,495 (0.5) | 7,338 (0.2) | 3,588 (0.1) | 4,559 (0.1) |
| **2012** | 172,375 (3.7) | 36,457 (0.8) | 16,108 (0.3) | 8,189 (0.2) | 25,637 (0.6) | 7,089 (0.2) | 3,988 (0.1) | 5,179 (0.1) |
| **2014** | 194,701 (4.2) | 38,894 (0.8) | 19,130 (0.4) | 8,387 (0.2) | 26,947 (0.6) | 6,641 (0.1) | 4,532 (0.1) | 6,067 (0.1) |
| **2016** | 185,653 (4.0) | 35,163 (0.8) | 18,419 (0.4) | 7,408 (0.2) | 27,280 (0.6) | 6,508 (0.1) | 4,870 (0.1) | 5,939 (0.1) |
| **2018** | 182,506 (3.9) | 31,563 (0.7) | 18,391 (0.4) | 7,541 (0.2) | 29,031 (0.6) | 6,782 (0.1) | 5,268 (0.1) | 6,609 (0.1) |
| **2020** | 88,311 (1.9) | 13,998 (0.3) | 8,924 (0.2) | 3,504 (0.1) | 15,636 (0.3) | 3,475 (0.1) | 2,999 (0.1) | 3,662 (0.1) |
| **Total** | 1,473,042 (31.7) | 322,523 ( 6.9) | 141,379 ( 3.0) | 72,567 ( 1.6) | 230,191 ( 5.0) | 65,914 ( 1.4) | 47,545 ( 1.0) | 34,985 ( 0.8) |
