# Supplementary material for: Time-varying exposure to food retailers and cardiovascular disease hospitalization and mortality in the netherlands: a nationwide prospective cohort study
Source: BMC Med. 2024 Oct 8;22:427. doi: 10.1186/s12916-024-03648-w (PMC11462997; doi:10.1186/s12916-024-03648-w)
Supplement: Supplementary file 8 — Additional file 8. Hazard Ratios and confidence intervals for Hospitalization for general and specific cardiovascular events in relation to longitudinal exposure to neighborhood food environment or food stores in a 1000 to meter buffer. [file 12916_2024_3648_MOESM8_ESM.docx]

**Additional files of ‘Time-varying exposure to food retailers and cardiovascular disease hospitalization and mortality in the Netherlands: A nationwide prospective cohort study**

**Additional file 8.** Hazard Ratios and confidence intervals for Hospitalization for general and specific cardiovascular events in relation to longitudinal exposure to neighborhood food environment or food stores in a 1000 to meter buffer.

|  | CVD Hospitalization | | CHD Hospitalization | | Stroke Hospitalization | | Heart failure Hospitalization | |
| --- | --- | --- | --- | --- | --- | --- | --- | --- |
|  | HR | 95% CI | HR | 95% CI | HR | 95% CI | HR | 95% CI |
| FEHI | 0.902 | 0.886 to 0.908 | 0.879 | 0.846 to 0.907 | 0.889 | 0.835 to 0.930 | 0.900 | 0.840 to 0.957 |
| Local food shops | 1.002 | 1.001 to 1.002 | 1.002 | 1.001 to 1.004 | 1.005 | 1.003 to 1.008 | 1.010 | 1.007 to 1.013 |
| Fast food outlets | 1.003 | 1.002 to 1.004 | 1.005 | 1.004 to 1.007 | 1.007 | 1.005 to 1.009 | 1.012 | 1.009 to 1.015 |
| Food delivery outlets | 0.996 | 0.995 to 0.996 | 0.995 | 0.994 to 0.996 | 0.999 | 0.997 to 1.000 | 0.998 | 0.996 to 1.000 |
| Restaurants | 0.998 | 0.998 to 0.998 | 0.997 | 0.997 to 0.998 | 0.999 | 0.999 to 1.000 | 0.999 | 0.998 to 0.999 |
| Supermarkets | 1.010 | 1.007 to 1.012 | 1.017 | 1.012 to 1.021 | 1.014 | 1.008 to 1.020 | 1.039 | 1.031 to 1.048 |
| Convenience stores | 1.004 | 1.003 to 1.006 | 1.005 | 1.002 to 1.009 | 1.001 | 0.997 to 1.006 | 1.020 | 1.014 to 1.026 |

*Models were adjusted for age, sex, ethnicity, household composition, household income, marital status, and neighborhood urbanization levels.

FEHI = food environment healthiness index
