# Supplementary material for: Time-varying exposure to food retailers and cardiovascular disease hospitalization and mortality in the netherlands: a nationwide prospective cohort study
Source: BMC Med. 2024 Oct 8;22:427. doi: 10.1186/s12916-024-03648-w (PMC11462997; doi:10.1186/s12916-024-03648-w)
Supplement: Supplementary file 9 — Additional file 9. Hazard Ratios and confidence intervals for general and specific cardiovascular mortality in relation to longitudinal exposure to neighborhood food environment or food stores in a 1000 to meter buffer. [file 12916_2024_3648_MOESM9_ESM.docx]

**Additional files of ‘Time-varying exposure to food retailers and cardiovascular disease hospitalization and mortality in the Netherlands: A nationwide prospective cohort study**

**Additional file 9.** Hazard Ratios and confidence intervals for general and specific cardiovascular mortality in relation to longitudinal exposure to neighborhood food environment or food stores in a 1000 to meter buffer.

|  | CVD mortality | | CHD mortality | | Stroke mortality | | Heart Failure mortality | |
| --- | --- | --- | --- | --- | --- | --- | --- | --- |
|  | HR | 95% CI | HR | 95% CI | HR | 95% CI | HR | 95% CI |
| FEHI | 0.847 | 0.823 to 0.890 | 0.802 | 0.754 to 0.863 | 0.889 | 0.824 to 0.977 | 0.843 | 0.759 to 0.916 |
| Local food shops | 1.007 | 1.005 to 1.009 | 1.008 | 1.005 to 1.011 | 1.005 | 1.002 to 1.009 | 1.010 | 1.001 to 1.011 |
| Fast food outlets | 1.008 | 1.006 to 1.010 | 1.013 | 1.010 to 1.015 | 1.002 | 0.999 to 1.006 | 1.007 | 1.003 to 1.011 |
| Food delivery outlets | 0.997 | 0.996 to 0.999 | 0.998 | 0.996 to 1.000 | 0.999 | 0.996 to 1.001 | 0.993 | 0.990 to 0.996 |
| Restaurants | 0.998 | 0.998 to 0.999 | 0.998 | 0.997 to 0.999 | 0.998 | 0.997 to 0.999 | 0.999 | 0.997 to 1.000 |
| Supermarkets | 1.022 | 1.017 to 1.027 | 1.034 | 1.025 to 1.044 | 1.006 | 0.995 to 1.016 | 1.022 | 1.010 to 1.035 |
| Convenience stores | 1.011 | 1.010 to 1.015 | 1.023 | 1.016 to 1.029 | 1.005 | 0.997 to 1.013 | 1.004 | 0.995 to 1.014 |

*Models were adjusted for age, sex, ethnicity, household composition, household income, marital status, and neighborhood urbanization levels.

FEHI = food environment healthiness index
