# Supplementary material for: Time-varying exposure to food retailers and cardiovascular disease hospitalization and mortality in the netherlands: a nationwide prospective cohort study
Source: BMC Med. 2024 Oct 8;22:427. doi: 10.1186/s12916-024-03648-w (PMC11462997; doi:10.1186/s12916-024-03648-w)
Supplement: Supplementary file 11 — Additional file 11. Hazard Ratios and confidence intervals for general and specific cardiovascular mortality in relation to longitudinal exposure to neighborhood food environment – models additionally adjusted for the broader food environment. [file 12916_2024_3648_MOESM11_ESM.docx]

**Additional files of ‘Time-varying exposure to food retailers and cardiovascular disease hospitalization and mortality in the Netherlands: A nationwide prospective cohort study**

**Additional file 11.** Hazard Ratios and confidence intervals for general and specific cardiovascular mortality in relation to longitudinal exposure to neighborhood food environment – **models additionally adjusted for the broader food environment**.

|  | CVD mortality | | CHD mortality | | Stroke mortality | | Heart Failure mortality | |
| --- | --- | --- | --- | --- | --- | --- | --- | --- |
|  | HR | 95% CI | HR | 95% CI | HR | 95% CI | HR | 95% CI |
| Local food shops | 1.012 | 1.011 to 1.015 | 1.013 | 1.009 to 1.017 | 1.013 | 1.008 to 1.018 | 1.012 | 1.006 to 1.018 |
| Fast food outlets | 1.030 | 1.027 to 1.033 | 1.044 | 1.038 to 1.049 | 1.022 | 1.013 to 1.024 | 1.032 | 1.021 to 1.042 |
| Food delivery outlets | 0.995 | 0.994 to 0.997 | 0.995 | 0.992 to 0.998 | 0.999 | 0.996 to 1.003 | 0.988 | 0.984 to 0.992 |
| Restaurants | 0.993 | 0.992 to 0.994 | 0.991 | 0.989 to 0.993 | 0.995 | 0.993 to 0.997 | 0.995 | 0.993 to 0.997 |
| Supermarkets | 1.031 | 1.025 to 1.038 | 1.045 | 1.034 to 1.056 | 1.013 | 1.001 to 1.026 | 1.034 | 1.019 to 1.049 |
| Convenience stores | 1.016 | 1.012 to 1.020 | 1.030 | 1.022 to 1.037 | 1.010 | 1.002 to 1.019 | 1.007 | 0.996 to 1.018 |

*Models were adjusted for age, sex, ethnicity, household composition, household income, marital status, and neighborhood urbanization levels.

FEHI = food environment healthiness index is not included in this table because the index by definition accounts for a variety of food outlets in the neighborhood
