# Supplementary material for: Time-varying exposure to food retailers and cardiovascular disease hospitalization and mortality in the netherlands: a nationwide prospective cohort study
Source: BMC Med. 2024 Oct 8;22:427. doi: 10.1186/s12916-024-03648-w (PMC11462997; doi:10.1186/s12916-024-03648-w)
Supplement: Supplementary file 12 — Additional file 12. Hazard Ratios and confidence intervals for Hospitalization of general and specific cardiovascular events in relation to longitudinal exposure to neighborhood food environment – analyses stratified by neighborhood urbanization. [file 12916_2024_3648_MOESM12_ESM.docx]

**Additional files of ‘Time-varying exposure to food retailers and cardiovascular disease hospitalization and mortality in the Netherlands: A nationwide prospective cohort study**

**Additional file 12**. Hazard Ratios and confidence intervals for Hospitalization of general and specific cardiovascular events in relation to longitudinal exposure to neighborhood food environment – **analyses stratified by neighborhood urbanization**.

| **Very high urbanization**  **N = 843,266** | | | | | | | | |
| --- | --- | --- | --- | --- | --- | --- | --- | --- |
|  | **CVD Hospitalization** | | **CHD Hospitalization** | | **Stroke Hospitalization** | | **Heart Failure Hospitalization** | |
|  | HR | 95% CI | HR | 95% CI | HR | 95% CI | HR | 95% CI |
| FEHI | 0.799 | 0.726 to 0.879 | 0.794 | 0.647 to 0.975 | 0.520 | 0.390 to 0.694 | 0.374 | 0.264 to 0.531 |
| Local food shops | 0.998 | 0.997 to 0.999 | 0.998 | 0.996 to 0.999 | 1.000 | 0.997 to 1.003 | 1.003 | 1.000 to 1.007 |
| Fast food outlets | 0.999 | 0.998 to 0.999 | 0.999 | 0.997 to 1.000 | 1.003 | 1.000 to 1.005 | 1.006 | 1.003 to 1.010 |
| Food delivery outlets | 0.996 | 0.996 to 0.997 | 0.996 | 0.995 to 0.997 | 0.999 | 0.997 to 1.000 | 0.998 | 0.996 to 1.001 |
| Restaurants | 0.998 | 0.997 to 0.998 | 0.997 | 0.996 to 0.998 | 0.999 | 0.998 to 0.999 | 0.998 | 0.997 to 0.999 |
| Supermarkets | 0.994 | 0.991 to 0.998 | 0.996 | 0.990 to 1.003 | 1.002 | 0.992 to 1.012 | 1.012 | 0.999 to 1.025 |
| Convenience stores | 1.003 | 1.001 to 1.004 | 1.002 | 0.999 to 1.006 | 0.998 | 0.994 to 1.003 | 1.017 | 1.011 to 1.023 |
| **High urbanization**  **N = 1,180,651** | | | | | | | | |
|  | **CVD Hospitalization** | | **CHD Hospitalization** | | **Stroke Hospitalization** | | **Heart Failure Hospitalization** | |
|  | HR | 95% CI | HR | 95% CI | HR | 95% CI | HR | 95% CI |
| FEHI | 0.716 | 0.679 to 0.775 | 0.707 | 0.634 to 0.789 | 0.743 | 0.632 to 0.873 | 0.642 | 0.519 to 0.795 |
| Local food shops | 1.003 | 1.002 to 1.005 | 1.005 | 1.002 to 1.009 | 1.009 | 1.004 to 1.014 | 1.016 | 1.009 to 1.022 |
| Fast food outlets | 1.012 | 1.010 to 1.013 | 1.022 | 1.019 to 1.016 | 1.014 | 1.009 to 1.020 | 1.026 | 1.019 to 1.033 |
| Food delivery outlets | 0.992 | 0.990 to 0.994 | 0.988 | 0.984 to 0.993 | 0.999 | 0.992 to 1.006 | 1.005 | 0.997 to 1.014 |
| Restaurants | 0.998 | 0.997 to 0.998 | 0.999 | 0.997 to 1.001 | 1.001 | 0.999 to 1.004 | 1.002 | 0.999 to 1.005 |
| Supermarkets | 1.023 | 1.018 to 1.027 | 1.034 | 1.025 to 1.043 | 1.027 | 1.014 to 1.040 | 1.067 | 1.050 to 1.085 |
| Convenience stores | 1.019 | 1.013 to 1.026 | 1.039 | 1.025 to 1.053 | 1.030 | 1.010 to 1.051 | 1.099 | 1.071 to 1.128 |
| **Moderate urbanization**  **N = 900,219** | | | | | | | | |
|  | **CVD Hospitalization** | | **CHD Hospitalization** | | **Stroke Hospitalization** | | **Heart Failure Hospitalization** | |
|  | HR | 95% CI | HR | 95% CI | HR | 95% CI | HR | 95% CI |
| FEHI | 0.796 | 0.756 to 0.838 | 0.788 | 0.711 to 0.873 | 0.813 | 0.690 to 0.957 | 0.775 | 0.621 to 0.968 |
| Local food shops | 1.006 | 1.004 to 1.009 | 1.002 | 0.998 to 1.007 | 1.013 | 1.006 to 1.020 | 1.031 | 1.021 to 1.041 |
| Fast food outlets | 1.018 | 1.014 to 1.021 | 1.026 | 1.019 to 1.033 | 1.020 | 1.010 to 1.031 | 1.043 | 1.029 to 1.058 |
| Food delivery outlets | 1.005 | 1.001 to 1.010 | 0.998 | 0.990 to 1.007 | 0.994 | 0.982 to 1.007 | 0.996 | 0.977 to 1.014 |
| Restaurants | 1.000 | 0.999 to 1.002 | 0.998 | 0.995 to 1.001 | 1.002 | 0.998 to 1.006 | 1.007 | 1.003 to 1.012 |
| Supermarkets | 1.019 | 1.014 to 1.024 | 1.018 | 1.008 to 1.029 | 1.027 | 1.011 to 1.043 | 1.069 | 1.046 to 1.092 |
| Convenience stores | 1.058 | 1.044 to 1.073 | 1.059 | 1.030 to 1.090 | 1.085 | 1.042 to 1.131 | 1.099 | 1.038 to 1.164 |
| **Low urbanization**  **N = 815,115** | | | | | | | | |
|  | **CVD Hospitalization** | | **CHD Hospitalization** | | **Stroke Hospitalization** | | **Heart Failure Hospitalization** | |
|  | HR | 95% CI | HR | 95% CI | HR | 95% CI | HR | 95% CI |
| FEHI | 0.887 | 0.849 to 0.926 | 0.849 | 0.775 to 0.931 | 0.905 | 0.789 to 1.039 | 0.826 | 0.691 to 0.988 |
| Local food shops | 1.011 | 1.008 to 1.015 | 1.013 | 1.007 to 1.020 | 1.011 | 1.001 to 1.021 | 1.032 | 1.019 to 1.046 |
| Fast food outlets | 1.033 | 1.028 to 1.038 | 1.043 | 1.034 to 1.053 | 1.030 | 1.016 to 1.045 | 1.060 | 1.039 to 1.081 |
| Food delivery outlets | 1.006 | 0.998 to 1.014 | 0.986 | 0.970 to 1.003 | 1.012 | 0.987 to 1.038 | 1.014 | 0.978 to 1.051 |
| Restaurants | 0.997 | 0.995 to 1.000 | 0.998 | 0.994 to 1.003 | 0.995 | 0.989 to 1.001 | 1.004 | 0.995 to 1.012 |
| Supermarkets | 1.031 | 1.025 to 1.038 | 1.036 | 1.023 to 1.050 | 1.018 | 0.998 to 1.038 | 1.087 | 1.058 to 1.116 |
| Convenience stores | 1.067 | 1.043 to 1.091 | 1.171 | 1.121 to 1.224 | 1.070 | 0.997 to 1.148 | 1.001 | 0.907 to 1.105 |
| **No urbanization**  **N = 902,184** | | | | | | | | |
|  | **CVD Hospitalization** | | **CHD Hospitalization** | | **Stroke Hospitalization** | | **Heart Failure Hospitalization** | |
|  | HR | 95% CI | HR | 95% CI | HR | 95% CI | HR | 95% CI |
| FEHI | 0.960 | 0.940 to 0.980 | 0.934 | 0.895 to 0.976 | 0.950 | 0.889 to 1.016 | 1.058 | 0.954 to 1.173 |
| Local food shops | 1.027 | 1.021 to 1.032 | 1.051 | 1.040 to 1.062 | 1.011 | 0.994 to 1.028 | 0.983 | 0.960 to 1.007 |
| Fast food outlets | 1.046 | 1.039 to 1.054 | 1.082 | 1.067 to 1.099 | 1.026 | 1.002 to 1.050 | 1.002 | 0.969 to 1.036 |
| Food delivery outlets | 1.015 | 0.998 to 1.032 | 1.008 | 0.973 to 1.045 | 1.047 | 0.994 to 1.103 | 0.935 | 0.863 to 1.014 |
| Restaurants | 1.000 | 0.997 to 1.002 | 1.006 | 1.000 to 1.013 | 0.995 | 0.985 to 1.005 | 0.982 | 0.967 to 0.997 |
| Supermarkets | 1.060 | 1.049 to 1.070 | 1.103 | 1.081 to 1.126 | 1.028 | 0.997 to 1.061 | 0.998 | 0.954 to 1.043 |
| Convenience stores | 1.000 | 0.976 to 1.025 | 1.034 | 0.985 to 1.086 | 0.924 | 0.853 to 1.001 | 0.944 | 0.845 to 1.054 |

*Models were adjusted for age, sex, ethnicity, household composition, household income and marital status.

FEHI = food environment healthiness index
