# Supplementary material for: Time-varying exposure to food retailers and cardiovascular disease hospitalization and mortality in the netherlands: a nationwide prospective cohort study
Source: BMC Med. 2024 Oct 8;22:427. doi: 10.1186/s12916-024-03648-w (PMC11462997; doi:10.1186/s12916-024-03648-w)
Supplement: Supplementary file 13 — Additional file 13. Hazard Ratios and confidence intervals for general and specific cardiovascular Hospitalization in relation to longitudinal exposure to neighborhood food environment – analyses stratified by sex. [file 12916_2024_3648_MOESM13_ESM.docx]

**Additional files of ‘Time-varying exposure to food retailers and cardiovascular disease hospitalization and mortality in the Netherlands: A nationwide prospective cohort study**

**Additional file 13**. Hazard Ratios and confidence intervals for general and specific cardiovascular Hospitalization in relation to longitudinal exposure to neighborhood food environment – **analyses stratified by sex**.

| **Female**  **N = 2,380,101** | | | | | | | | |
| --- | --- | --- | --- | --- | --- | --- | --- | --- |
|  | **CVD Hospitalization** | | **CHD Hospitalization** | | **Stroke Hospitalization** | | **Heart Failure Hospitalization** | |
|  | HR | 95% CI | HR | 95% CI | HR | 95% CI | HR | 95% CI |
| FEHI index | 0.903 | 0.882 to 0.925 | 0.873 | 0.824 to 0.925 | 0.913 | 0.846 to 0.986 | 0.898 | 0.819 to 0.985 |
| Local food shops | 1.002 | 1.001 to 1.003 | 1.005 | 1.002 to 1.007 | 1.005 | 1.002 to 1.008 | 1.010 | 1.006 to 1.014 |
| Fast food outlets | 1.003 | 1.002 to 1.004 | 1.007 | 1.006 to 1.010 | 1.006 | 1.003 to 1.009 | 1.012 | 1.009 to 1.016 |
| Food delivery outlets | 0.995 | 0.994 to 0.996 | 0.996 | 0.994 to 0.998 | 0.998 | 0.996 to 1.001 | 0.998 | 0.995 to 1.001 |
| Restaurants | 0.997 | 0.997 to 0.997 | 0.997 | 0.996 to 0.998 | 0.999 | 0.998 to 1.000 | 0.998 | 0.997 to 0.999 |
| Supermarkets | 1.012 | 1.009 to 1.015 | 1.021 | 1.014 to 1.028 | 1.013 | 1.004 to 1.022 | 1.037 | 1.026 to 1.050 |
| Convenience stores | 1.007 | 1.004 to 1.009 | 1.011 | 1.006 to 1.016 | 1.002 | 0.995 to 1.008 | 1.025 | 1.017 to 1.033 |
| **Male**  **N = 2,261,334** | | | | | | | | |
|  | **CVD Hospitalization** | | **CHD Hospitalization** | | **Stroke Hospitalization** | | **Heart Failure Hospitalization** | |
|  | HR | 95% CI | HR | 95% CI | HR | 95% CI | HR | 95% CI |
| FEHI index | 0.898 | 0.879 to 0.918 | 0.884 | 0.849 to 0.920 | 0.868 | 0.812 to 0.928 | 0.913 | 0.820 to 1.016 |
| Local food shops | 1.001 | 1.000 to 1.002 | 1.001 | 0.999 to 1.003 | 1.006 | 1.003 to 1.009 | 1.010 | 1.006 to 1.014 |
| Fast food outlets | 1.002 | 1.001 to 1.003 | 1.004 | 1.002 to 1.006 | 1.007 | 1.004 to 1.010 | 1.011 | 1.007 to 1.015 |
| Food delivery outlets | 0.996 | 0.996 to 0.997 | 0.995 | 0.993 to 0.996 | 0.999 | 0.997 to 1.001 | 0.998 | 0.995 to 1.001 |
| Restaurants | 0.998 | 0.998 to 0.998 | 0.997 | 0.997 to 0.998 | 1.000 | 0.999 to 1.001 | 0.999 | 0.998 to 1.000 |
| Supermarkets | 1.008 | 1.005 to 1.011 | 1.014 | 1.008 to 1.019 | 1.014 | 1.005 to 1.023 | 1.039 | 1.026 to 1.052 |
| Convenience stores | 1.002 | 1.000 to 1.004 | 1.003 | 0.999 to 1.007 | 1.001 | 0.995 to 1.008 | 1.013 | 1.004 to 1.021 |

*Models were adjusted for age, ethnicity, household composition, household income, marital status, and neighborhood urbanization levels.

FEHI = food environment healthiness index
