# Supplementary material for: Time-varying exposure to food retailers and cardiovascular disease hospitalization and mortality in the netherlands: a nationwide prospective cohort study
Source: BMC Med. 2024 Oct 8;22:427. doi: 10.1186/s12916-024-03648-w (PMC11462997; doi:10.1186/s12916-024-03648-w)
Supplement: Supplementary file 14 — Additional file 14. Hazard Ratios and confidence intervals for Hospitalization of general and specific cardiovascular events in relation to longitudinal exposure to neighborhood food environment – analyses stratified by age. [file 12916_2024_3648_MOESM14_ESM.docx]

**Additional files of ‘Time-varying exposure to food retailers and cardiovascular disease hospitalization and mortality in the Netherlands: A nationwide prospective cohort study**

**Additional file 14**. Hazard Ratios and confidence intervals for Hospitalization of general and specific cardiovascular events in relation to longitudinal exposure to neighborhood food environment – **analyses stratified by age**.

| **Age 35 – 49**  **N = 1,841,486** | | | | | | | | |
| --- | --- | --- | --- | --- | --- | --- | --- | --- |
|  | **CVD Hospitalization** | | **CHD Hospitalization** | | **Stroke Hospitalization** | | **Heart Failure Hospitalization** | |
|  | HR | 95% CI | HR | 95% CI | HR | 95% CI | HR | 95% CI |
| FEHI | 0.871 | 0.841 to 0.901 | 0.812 | 0.758 to 0.869 | 0.854 | 0.762 to 0.957 | 0.923 | 0.686 to 1.242 |
| Local food shops | 0.998 | 0.996 to 0.999 | 0.997 | 0.994 to 1.001 | 1.009 | 1.003 to 1.014 | 1.007 | 0.996 to 1.018 |
| Fast food outlets | 1.001 | 1.000 to 1.003 | 1.003 | 1.000 to 1.006 | 1.012 | 1.008 to 1.017 | 1.009 | 0.999 to 1.019 |
| Food delivery outlets | 0.991 | 0.990 to 0.993 | 0.990 | 0.988 to 0.992 | 0.997 | 0.994 to 1.001 | 0.993 | 0.987 to 1.000 |
| Restaurants | 0.995 | 0.994 to 0.996 | 0.994 | 0.992 to 0.995 | 0.998 | 0.996 to 0.999 | 0.995 | 0.991 to 0.999 |
| Supermarkets | 1.018 | 1.013 to 1.022 | 1.015 | 1.005 to 1.024 | 1.030 | 1.014 to 1.046 | 1.060 | 1.028 to 1.094 |
| Convenience stores | 1.002 | 0.999 to 1.005 | 1.003 | 0.997 to 1.010 | 1.013 | 1.003 to 1.024 | 1.011 | 0.991 to 1.032 |
| **Age 50 – 64**  **N = 1,792,280** | | | | | | | | |
|  | **CVD Hospitalization** | | **CHD Hospitalization** | | **Stroke Hospitalization** | | **Heart Failure Hospitalization** | |
|  | HR | 95% CI | HR | 95% CI | HR | 95% CI | HR | 95% CI |
| FEHI | 0.873 | 0.852 to 0.894 | 0.886 | 0.843 to 0.932 | 0.848 | 0.786 to 0.915 | 0.839 | 0.734 to 0.959 |
| Local food shops | 1.001 | 1.001 to 1.003 | 1.002 | 0.999 to 1.004 | 1.005 | 1.001 to 1.009 | 1.011 | 1.005 to 1.017 |
| Fast food outlets | 1.003 | 1.002 to 1.004 | 1.005 | 1.003 to 1.007 | 1.007 | 1.004 to 1.010 | 1.011 | 1.006 to 1.016 |
| Food delivery outlets | 0.995 | 0.994 to 0.995 | 0.995 | 0.994 to 0.997 | 0.997 | 0.995 to 0.999 | 0.995 | 0.991 to 0.999 |
| Restaurants | 0.997 | 0.997 to 0.998 | 0.997 | 0.996 to 0.998 | 0.999 | 0.998 to 1.000 | 0.997 | 0.995 to 0.999 |
| Supermarkets | 1.019 | 1.016 to 1.022 | 1.023 | 1.017 to 1.030 | 1.028 | 1.017 to 1.038 | 1.045 | 1.027 to 1.064 |
| Convenience stores | 1.006 | 1.004 to 1.008 | 1.005 | 1.001 to 1.010 | 1.002 | 0.994 to 1.009 | 1.020 | 1.008 to 1.031 |
| **Age ≥ 65**  **N = 1,007,669** | | | | | | | | |
|  | **CVD Hospitalization** | | **CHD Hospitalization** | | **Stroke Hospitalization** | | **Heart Failure Hospitalization** | |
|  | HR | 95% CI | HR | 95% CI | HR | 95% CI | HR | 95% CI |
| FEHI | 0.924 | 0.901 to 0.948 | 0.928 | 0.877 to 0.982 | 0.954 | 0.880 to 1.035 | 0.915 | 0.842 to 0.995 |
| Local food shops | 1.005 | 1.004 to 1.006 | 1.004 | 1.002 to 1.006 | 1.003 | 1.000 to 1.006 | 1.014 | 1.011 to 1.017 |
| Fast food outlets | 1.003 | 1.002 to 1.004 | 1.005 | 1.003 to 1.008 | 1.001 | 0.998 to 1.004 | 1.011 | 1.008 to 1.014 |
| Food delivery outlets | 0.999 | 0.998 to 0.999 | 0.997 | 0.996 to 0.999 | 0.999 | 0.997 to 1.001 | 0.998 | 0.996 to 1.001 |
| Restaurants | 0.999 | 0.999 to 0.999 | 0.999 | 0.998 to 0.999 | 1.000 | 0.998 to 1.002 | 1.000 | 0.999 to 1.001 |
| Supermarkets | 1.008 | 1.005 to 1.011 | 1.007 | 1.000 to 1.014 | 0.995 | 0.986 to 1.005 | 1.045 | 1.035 to 1.056 |
| Convenience stores | 1.002 | 0.999 to 1.004 | 1.001 | 0.996 to 1.007 | 0.987 | 0.980 to 0.995 | 1.014 | 1.007 to 1.022 |

*Models were adjusted for sex, ethnicity, household composition, household income, marital status, and neighborhood urbanization levels.

FEHI = food environment healthiness index
