# Supplementary material for: Time-varying exposure to food retailers and cardiovascular disease hospitalization and mortality in the netherlands: a nationwide prospective cohort study
Source: BMC Med. 2024 Oct 8;22:427. doi: 10.1186/s12916-024-03648-w (PMC11462997; doi:10.1186/s12916-024-03648-w)
Supplement: Supplementary file 15 — Additional file 15. Hazard Ratios and confidence intervals for Hospitalization of general and specific cardiovascular events in relation to longitudinal exposure to neighborhood food environment – analyses stratified by levels of household income. [file 12916_2024_3648_MOESM15_ESM.docx]

**Additional files of ‘Time-varying exposure to food retailers and cardiovascular disease hospitalization and mortality in the Netherlands: A nationwide prospective cohort study**

**Additional file 15**. Hazard Ratios and confidence intervals for Hospitalization of general and specific cardiovascular events in relation to longitudinal exposure to neighborhood food environment – **analyses stratified by levels of household income**.

| **Low income**  **(Median 15,346, IQR 13,597 to 16,614)**  **N = 464,268** | | | | | | | | |
| --- | --- | --- | --- | --- | --- | --- | --- | --- |
|  | **CVD Hospitalization** | | **CHD Hospitalization** | | **Stroke Hospitalization** | | **Heart Failure Hospitalization** | |
|  | HR | 95% CI | HR | 95% CI | HR | 95% CI | HR | 95% CI |
| FEHI | 0.929 | 0.883 to 0.978 | 0.965 | 0.858 to 1.088 | 0.903 | 0.770 to 1.059 | 1.107 | 0.874 to 1.401 |
| Local food shops | 0.994 | 0.993 to 0.996 | 0.996 | 0.993 to 0.999 | 0.997 | 0.992 to 1.002 | 0.998 | 0.991 to 1.006 |
| Fast food outlets | 0.996 | 0.995 to 0.998 | 0.999 | 0.996 to 1.003 | 0.997 | 0.992 to 1.002 | 1.002 | 0.995 to 1.009 |
| Food delivery outlets | 0.995 | 0.994 to 0.996 | 0.996 | 0.993 to 0.998 | 0.999 | 0.996 to 1.002 | 0.994 | 0.989 to 0.998 |
| Restaurants | 0.996 | 0.996 to 0.997 | 0.997 | 0.996 to 0.998 | 0.998 | 0.997 to 0.999 | 0.995 | 0.993 to 0.997 |
| Supermarkets | 0.994 | 0.989 to 0.999 | 1.008 | 0.997 to 1.019 | 1.000 | 0.984 to 1.016 | 0.999 | 0.978 to 1.022 |
| Convenience stores | 0.998 | 0.995 to 1.001 | 1.003 | 0.997 to 1.009 | 0.981 | 0.972 to 0.991 | 1.004 | 0.990 to 1.018 |
| **Middle income**  **(Median 28,757, IQR 23,270 to 35,087)**  **N = 3,713,058** | | | | | | | | |
|  | **CVD Hospitalization** | | **CHD Hospitalization** | | **Stroke Hospitalization** | | **Heart Failure Hospitalization** | |
|  | HR | 95% CI | HR | 95% CI | HR | 95% CI | HR | 95% CI |
| FEHI | 0.914 | 0.898 to 0.931 | 0.888 | 0.857 to 0.921 | 0.898 | 0.849 to 0.949 | 0.898 | 0.832 to 0.970 |
| Local food shops | 1.003 | 1.002 to 1.004 | 1.003 | 1.001 to 1.005 | 1.007 | 1.004 to 1.009 | 1.012 | 1.009 to 1.016 |
| Fast food outlets | 1.004 | 1.003 to 1.004 | 1.006 | 1.005 to 1.008 | 1.008 | 1.006 to 1.010 | 1.013 | 1.010 to 1.016 |
| Food delivery outlets | 0.996 | 0.995 to 0.996 | 0.995 | 0.994 to 0.997 | 0.998 | 0.996 to 1.000 | 0.999 | 0.997 to 1.002 |
| Restaurants | 0.998 | 0.998 to 0.998 | 0.997 | 0.997 to 0.998 | 1.000 | 0.999 to 1.000 | 0.999 | 0.998 to 1.001 |
| Supermarkets | 1.007 | 1.005 to 1.009 | 1.012 | 1.007 to 1.017 | 1.010 | 1.003 to 1.017 | 1.039 | 1.029 to 1.049 |
| Convenience stores | 1.005 | 1.003 to 1.007 | 1.005 | 1.001 to 1.008 | 1.005 | 0.999 to 1.010 | 1.023 | 1.017 to 1.030 |
| **High income**  **(Median 55,754, IQR 50,508 to 67,032)**  **N = 464,109** | | | | | | | | |
|  | **CVD Hospitalization** | | **CHD Hospitalization** | | **Stroke Hospitalization** | | **Heart Failure Hospitalization** | |
|  | HR | 95% CI | HR | 95% CI | HR | 95% CI | HR | 95% CI |
| FEHI | 0.841 | 0.791 to 0.895 | 0.802 | 0.708 to 0.908 | 0.882 | 0.732 to 1.063 | 0.714 | 0.542 to 0.940 |
| Local food shops | 1.005 | 1.002 to 1.008 | 1.009 | 1.004 to 1.015 | 1.013 | 1.004 to 1.022 | 1.019 | 1.008 to 1.031 |
| Fast food outlets | 1.002 | 1.000 to 1.005 | 1.004 | 0.999 to 1.009 | 1.012 | 1.005 to 1.020 | 1.016 | 1.007 to 1.025 |
| Food delivery outlets | 0.994 | 0.992 to 0.996 | 0.991 | 0.987 to 0.995 | 0.996 | 0.990 to 1.003 | 0.989 | 0.980 to 0.998 |
| Restaurants | 0.998 | 0.997 to 0.999 | 0.997 | 0.996 to 0.999 | 1.000 | 0.998 to 1.003 | 0.998 | 0.995 to 1.001 |
| Supermarkets | 1.010 | 1.002 to 1.019 | 1.026 | 1.009 to 1.044 | 1.018 | 0.992 to 1.045 | 1.094 | 1.058 to 1.132 |
| Convenience stores | 1.004 | 0.998 to 1.012 | 1.003 | 0.989 to 1.017 | 1.021 | 1.001 to 1.042 | 1.034 | 1.010 to 1.058 |

*Models were adjusted for age, sex, ethnicity, household composition, marital status, and neighborhood urbanization levels.

FEHI = food environment healthiness index
